# Association between Polymorphisms of the IKZF3 Gene and Systemic Lupus Erythematosus in a Chinese Han Population

**DOI:** 10.1371/journal.pone.0108661

**Published:** 2014-10-01

**Authors:** Xinze Cai, Ying Qiao, Cheng Diao, Xiaoxue Xu, Yang Chen, Shuyan Du, Xudong Liu, Nan Liu, Shuang Yu, Dong Chen, Yi Jiang

**Affiliations:** 1 Central Laboratory, First Affiliated Hospital of China Medical University, Shenyang, China; 2 Department of Immunology, College of Basic Medical Sciences, China Medical University, Shenyang, China; 3 Department of Dermatology, First Affiliated Hospital of China Medical University, Shenyang, China; Pavillon Kirmisson, France

## Abstract

**Objective:**

It has been reported that IKAROS family of zinc finger 3 (IKZF3)-deficient mice spontaneously develop human systemic lupus erythematosus (SLE)-like phenotypes and produce anti-dsDNA Ab leading to immune complex-mediated glomerulonephritis. Polymorphism of the IKZF3 gene corresponds with the susceptibility to several immune-related diseases. Our intention was to establish an association between polymorphisms in the IKZF3 gene and SLE in the Chinese Han population.

**Methods:**

The study involved obtaining blood samples for DNA extraction and genotyping the 4 selected single-nucleotide polymorphisms (SNPs) in IKZF3, including rs12150079, rs9909593, rs907091, and rs2872507, by performing PCR restriction fragment length polymorphism analysis (PCR-RFLP). A group of 366 SLE patients were compared to 455 healthy controls.

**Results:**

A significant decrease in frequencies of the rs907091 CC genotype and C allele appeared in the SLE patients unlike that observed in the controls (p = 0.001 and 0.015, respectively). The frequencies of the rs12150079 genotype and allele were different between the SLE patients and the control individuals, although the significance was only marginal (p = 0.046 and 0.049, respectively). In addition, a significantly low frequency of the GGCG haplotype was observed in the SLE patients, suggesting that it may provide protection against SLE (p = 0.011).

**Conclusion:**

To the best of our knowledge, this is the first study to demonstrate an important association between polymorphisms in IKZF3 and SLE in the Chinese Han population. A strong association between rs907091 in the IKZF3 gene and SLE was identified.

## Introduction

Systemic lupus erythematosus (SLE) is a chronic autoimmune disease with a wide spectrum of manifestations and severity. [1] Disease incidence is roughly 5.2 individuals per 100,000 persons per year and a higher incidence of SLE is seen among females, with a gender bias of roughly 9∶1. [2] The disease is caused by dysregulation of T cell responses and B cell activation, and is characterized by the formation of immune complexes which usually cause injuries of multiple organs and tissues such as skin, joints and kidneys. [3] Although it has been reported that SLE is affected by several factors including genetic and environmental ones, the pathogenesis of SLE has not been clarified clearly to date.

IKAROS family of zinc finger3 (IKZF3, also known as AIOLOS) encodes a zinc finger protein that is expressed at its highest levels in mature peripheral B cells and has chromatin remodeling and histone deacetylase activities through interacting with Ikaros proteins. [4], [5] In the periphery of mice homozygous for IKZF3-null mutation, B cells exhibit an activated cell surface phenotype and elevated serum IgG and IgE are detected in IKZF3-deficient mice. [6] It is demonstrated that IKZF3-deficient mice spontaneously develop human SLE-like phenotypes and produce anti-dsDNA Ab leading to immune complex-mediated glomerulonephritis. [7] With the development in genetic study, several single nucleotide polymorphisms (SNPs) in IKZF3 gene have been identified as being related to the susceptibility to immune-related diseases, including rheumatoid arthritis, ankylosing spondylitis, asthma and lupus. [3], [8]–[11] Association between SLE and variants in the region of IKZF3 has been recently identified in genome-wide association scan (GWAS) of SLE by testing a multiethnic population [11].

Therefore, in this study, we aimed to determine the association between IKZF3 polymorphisms and susceptibility to SLE in a Chinese population. In accordance with our aim, we analyzed 4 SNPs, including rs12150079, rs9909593, rs907091, and rs2872507.

## Methods

### Patients and Healthy Controls

366 patients with SLE diagnosed according to the criteria of the 1982 American College of Rheumatology were enrolled. [12] At the same time, 455 healthy controls without autoimmune disease or cancer were recruited, who were sex- and age-matched with the patients. The study participants were from the Chinese Han population and the age range was from 16 to 65.

### Ethics Statement

The Medical Ethics Committee of the First Affiliated Hospital of China Medical University approved the study. Written informed consent was obtained from all the participants, including the guardians on behalf of the children enrolled in our study.

### SNP Selection

Haplotype-tagging SNPs (tagSNPs) were selected using HapMap dbSNP (www.hapmap.ncbi.nlm.nih.gov) and Haploview (www. Broadinstitute.org/haploview/haploview), complemented by National Center for Biotechnology Information’s dbSNP (www.ncbi.nlm.nih.gov/projects/SNP), using LD-based tagSNP selection with a pairwise algorithm LDSelect available in Haploview (Tagger). [13], [14] A chromosomal region including IKZF3 gene and 4,000 bp upstream and 1,500 bp downstream (to capture the 5′ and 3′UTR) was searched, and all SNPs identified in Chinese Han population with a minor allele frequency (MAF) of 10% or greater were included in the algorithm. Among these tagSNPs, we selected rs12150079, rs9909593 and rs907091 as candidate SNPs. We also selected rs2872507 as a candidate SNP as it had been demonstrated earlier to be associated with RA [9].

### Genotyping

Genomic DNA was extracted via the conventional phenol-chloroform extraction method. Both the quality and quantity of the extracted DNA were then determined using spectrophotometry. The IKZF3 SNPs (rs12150079, rs9909593, rs907091, and rs2872507) were analyzed by restriction fragment length polymorphism (RFLP). [15] Primers used in PCR-RFLP were: for rs12150079, 5′-CATCTGCTTGGCAGTTCA-3′ (forward) and 5′-TGTTTCTGGTTTGGTGGA-3′ (reverse); for rs9909593, 5′-GCAGGAGGATTGATTG-3′ (forward) and 5′-ATTACCGCCACATTTA-3′ (reverse); for rs907091, 5′-TGTCATTTAGATTAGGGAGA-3′ (forward) and 5′-ACATAGCCAGAGGAGAAC-3′ (reverse); for rs2872507, 5′-TTTCCAAAATAAAGCAGTTC-3′ (forward) and 5′-ATGCCTAAAAGTAGCATCAA-3′ (reverse). The restriction enzymes for the SNPs above were Tth111 I, Blp I, Msp I and Nco I respectively (New England Biolabs). Direct sequencing was performed by using randomly selected subjects to validate the accuracy of the SNP genotyping assays (20% of the cases).

### Statistical analysis

Data were managed and stored using the SPSS software (version 16.0). Call rates were compared between SLE patient and control groups by the chi-square test. Hardy-Weinberg equilibrium (HWE) of the IKZF3 SNPs in the control subjects was evaluated using chi-square test. The frequencies of the alleles and genotypes were also compared between patient and control groups by the chi-square test. The odds ratio (OR) and 95% confidence intervals (95% CI) were estimated. The haplotypes comprising rs12150079, rs9909593, rs907091, and rs2872507 for each individual were assigned using the online software platform SHEsis (www.analysis2.bio-x.cn/myanalysis.php). All tests were two-tailed, and *p* values<0.05 were considered as statistically significant.

## Results

The 4 SNP (rs12150079, rs9909593, rs907091, and rs2872507) call rates in the patients and control individuals and their HWE p-values are shown in Table 1. The genotype frequencies of 4 SNPs were in agreement with the Hardy-Weinberg equilibrium in control group (p>0.05). There was no different concerning the call rates between the SLE group and the control (p>0.05).

**Table 1:** The 4 SNP call rates in patients and control individuals and HWE p-values.

SNP	Call rate (%)		HWE p-value	
	SLE	Control	SLE	Control
rs12150079	95.6%	98.9%	0.71	0.38
rs9909593	91.8%	92.7%	0.03	0.25
rs907091	92.1%	96.0%	0.01	0.68
rs2872507	94.8%	98.5%	0.18	0.27

The results of genotypic and allelic frequency analysis for the 4 SNPs in SLE patients and controls are shown in Table 2. With respect to the frequencies of rs907091, the SLE patients and the controls exhibited a significant difference. The frequency of the rs907091 CC genotype was much lower in SLE patients than in the controls, while the frequency of the rs907091 T allele was higher in patients (p = 0.001, odds ratio (OR) 0.38, 95% CI 0.21 to 0.68; p = 0.015, odds ratio (OR) 0.78, 95% CI 0.61 to 0.95, respectively). The results also show that the frequencies of the rs12150079 genotype and allele were different between the SLE patients and the control individuals, although the significance was only marginal (p = 0.046, odds ratio (OR) 1.78, 95% CI 1.01 to 3.14; p = 0.049, odds ratio (OR) 1.25, 95% CI 1.00 to 1.57, respectively). No difference in the genotype and allele frequencies in rs9909593 and rs2872507 was observed between the SLE and the control groups (p>0.05).

**Table 2:** Frequencies of alleles and genotypes of IKZF3 polymorphisms in SLE patients and control individuals.

SNP	Genotype	SLE (%)	Control (%)	χ^2^	P	Odds Ratio (95% CI)
	Allele	(N = 366)	(N = 455)			
rs12150079	GG	180 (51.4)	255 (56.7)			reference
	AG	140 (40.0)	172 (38.2)	1.11	0.292	1.17 (0.87–1.57)
	AA	30 (8.6)	23 (5.1)	3.99	0.046	1.78 (1.01–3.14)
	G	500 (71.4)	682 (75.8)			reference
	A	200 (28.6)	218 (24.2)	3.86	0.049	1.25 (1.00–1.57)
rs9909593	AA	123 (36.7)	175 (41.5)			reference
	AG	175 (52.2)	185 (43.8)	3.53	0.06	1.34 (0.99–1.84)
	GG	37 (11.0)	62 (14.7)	0.42	0.518	0.86 (0.54–1.37)
	A	421 (62.8)	535 (63.4)			reference
	G	249 (37.2)	309 (36.6)	0.05	0.825	0.97 (0.79–1.20)
rs907091	TT	168 (49.9)	197 (45.1)			reference
	CT	153 (45.4)	190 (43.5)	0.14	0.704	0.94 (0.70–1.27)
	CC	16 (4.7)	50 (11.4)	10.84	0.001	0.38 (0.21–0.68)
	T	489 (72.6)	584 (66.8)			reference
	C	185 (27.4)	290 (33.2)	5.87	0.015	0.76 (0.61–0.95)
rs2872507	GG	170 (49.0)	227 (50.7)			reference
	AG	153 (44.1)	177 (39.5)	0.92	0.339	1.15 (0.86–1.55)
	AA	24 (6.9)	44 (9.8)	1.35	0.245	0.73 (0.42–1.24)
	G	493 (71.0)	631 (70.4)			reference
	A	201 (29.0)	265 (29.6)	0.07	0.789	0.97 (0.78–1.21)

The online software platform SHEsis was used to analyze the haplotype data and probabilities in Table 3. Haplotypes were constructed in both SLE and control groups and the haplotypes with frequency of >1% were built from all the four SNPs. The results show that the GGCG haplotype frequency was significantly low in the SLE patients, unlike that observed in the controls (p = 0.011, odds ratio (OR) 0.42, 95% CI 0.21 to 0.84). In addition, a higher frequency of the GGTG haplotype was observed in the SLE patients than that in the controls (p = 0.027, odds ratio (OR) 2.10, 95% CI 1.07 to 4.12). No difference was detected between the SLE patients and the control individuals with respect the other 3 haplotypes (p>0.05).

**Table 3:** Frequencies of the haplotypes formed by rs12150079, rs9909593, rs907091 and rs2872507 SNPs in SLE patients and healthy control individuals.

Haplotype	SLE (%)	Control (%)	χ2	P	Odds Ratio (95% CI)
AGCA	101.13 (16.6)	168.69 (20.7)	1.43	0.231	0.85 (0.64–1.11)
GATG	363.19 (59.7)	507.62 (62.2)	1.15	0.283	1.14 (0.90–1.44)
GGCA	38.71 (6.4)	52.68 (6.5)	0.12	0.733	1.08 (0.70–1.66)
GGCG	10.81 (1.8)	36.42 (4.5)	6.42	0.011	0.42 (0.21–0.84)
GGTG	21.03 (3.5)	14.90 (1.8)	4.89	0.027	2.10 (1.07–4.12)

## Discussion

To our knowledge, this is the first report to determine the association between IKZF3 polymorphisms (rs12150079, rs9909593, rs907091 and rs2872507) and SLE susceptibility in a Chinese Han population. We confirmed that rs907091 and rs12150079 SNPs may have a relationship with SLE. The haplotype GGCG was found to protect against SLE, whereas the haplotype GGTG was associated with susceptibility to SLE.

SLE is prototypic, systemic, autoimmune disease, characterized by a diverse array of autoantibody production, complement activation and immune-complex deposition, and shows extremely heterogeneous clinical features varying in the severity. [3], [16] It appears that genetic factor is closely involved in the pathogenesis of SLE. Although the etiology of SLE remains incompletely understood, there is accumulating evidence showing the important role of IKZF3 in SLE [7], [11], [17].

Human IKZF3, which is mapped in chromosome 17, consists of 509 amino acid protein and forms homo or heterodimers with the Ikaros proteins in T and B lymphocytes. The physiological effect of IKZF3 deficiency is an increase in B cells precursors and spontaneous production of autoantibodies. [18] It was shown that B cell dysfunction, autoantibodies production, tissue injury, and SLE development existed in IKZF3-deficient mice. [7] Recently, multiple susceptibility loci of IKZF3 was successfully identified in case-control study of SLE [11].

**Figure 1:**
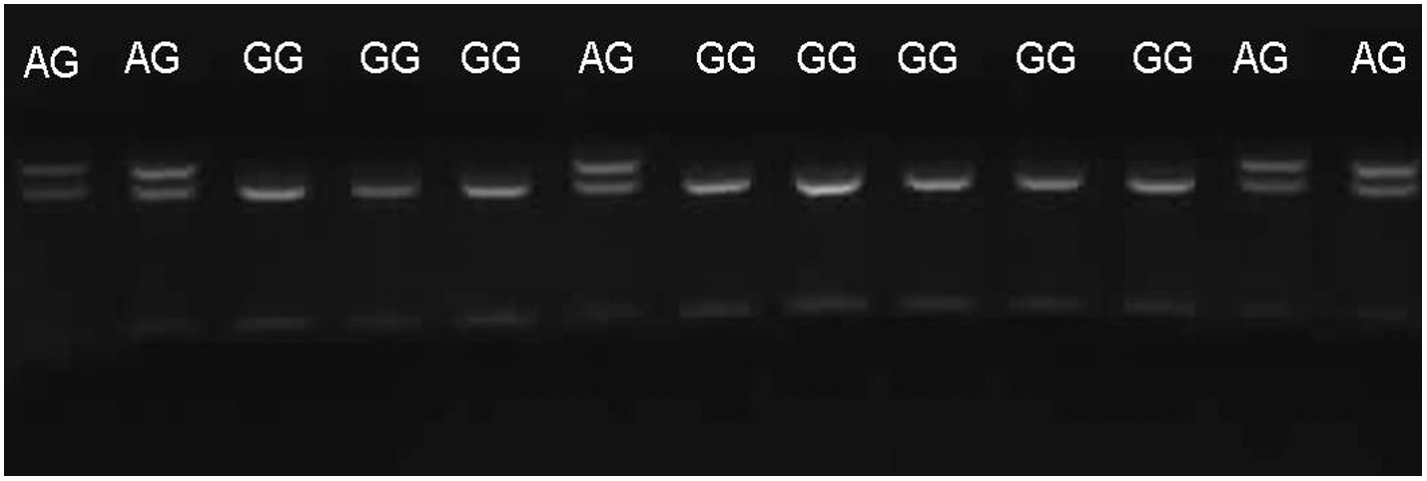
The representative results of the genotypes provided by PCR-RFLP and sequencing.

**Figure 2:**
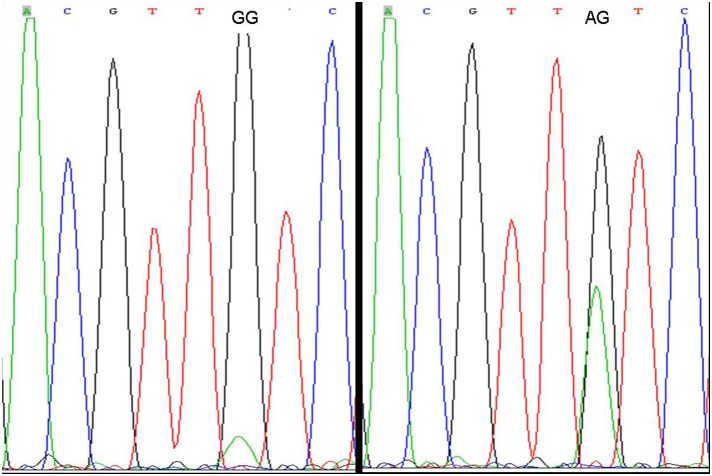
The representative results of the genotypes provided by PCR-RFLP and sequencing.

In the present study, we investigated the effect of 4 SNPs of the IKZF3 gene on SLE in Chinese Han population. Among the tagSNPs identified in Chinese Han population with MAF of 10% or greater, we selected rs12150079, rs9909593 and rs907091 as candidate SNPs; another polymorphism, rs2872507, was found to be associated with rheumatoid arthritis; [9] therefore, these SNPs were analyzed in our study. Considering that results on the association of gene polymorphisms with disease can be influenced by many factors, we aimed to ensure the correctness of our results. First, we selected SLE patients strictly according to the criteria of the 1982 American College of Rheumatology and excluded the ones who did not fit the criteria. [12] Second, we chose unrelated healthy individuals from the same geographic region as the controls; all the study participants were similar with respect to their age, sex and ethnicity. Finally, to verify the results of genotyping by PCR-RFLP, we repeated the sequencing of 20% of the samples. Direct sequencing was performed by using randomly selected 90 subjects (20% of the cases) to validate the accuracy of the SNP genotyping assays. Only 2 subjects failed in direct sequencing and the percentage of effective validation was close to 98% (Figure 1 and Figure 2). These approaches ensured the accuracy of the results of this study.

As stated in our study, the frequency of presence of the CC genotype and C allele of rs907091 in SLE patients (p = 0.001, odds ratio (OR) 0.38, 95% CI 0.21 to 0.68; p = 0.015, odds ratio (OR) 0.78, 95% CI 0.61 to 0.95) presented a significant decrease, suggesting a protective factor to SLE. Although the significance was only marginal, the frequency of presence of the AA genotype and A allele of rs12150079 in SLE patients (p = 0.046, odds ratio (OR) 1.78, 95% CI 1.01 to 3.14; p = 0.049, odds ratio (OR) 1.25, 95% CI 1.00 to 1.57) presented a moderate increase, indicating a potential susceptibility factor to this disease. Further studies on the influence of rs12150079 variants on SLE will require genetic analysis of a very large cohort. As different populations had different genetic backgrounds, which could cause ethnic heterogeneity, the genetic analysis of multiethnic study on the 4 SNPs will still be required. FASTSNP () is a web server that allows users to efficiently identify and prioritize high-risk SNPs according to their putative functional effects. We used FastSNP to predict the function for the different variants. We found that rs12150079 SNP might be a promoter in the regulatory region and rs9909593 might be an intronic enhancer.

Haplotype analysis revealed that the haplotype GGCG conferred a reduced risk of SLE, whereas GGTG was associated with susceptibility to SLE. By analyzing the results presented in Table 2 and Table 3, it is possible that the haplotype GGCG, which provides protection to SLE, results from the rs907091 C allele. As with other studies regarding the association of gene polymorphisms in SLE, our study still has limitations. This study only investigated SLE patients from a Chinese Han population. In addition, SLE is a heterogenetic disease and the environment is very important for the disease; gene-gene and gene-environment interactions are needed to clarify the impact of the gene on SLE. [19] Therefore, further research should be carried out in a larger number of samples and include analysis of the combined effect of multiple loci other than one single SNP or a single gene. Function analysis of the gene and the SNPs is also required to clarify the pathogenesis of SLE.

The previous published GWA scan of multiethnic population consisting of European and African American showed that rs9913957, rs8076347 and rs8079075 in IKZF3 gene contributed to the SLE susceptibility, and rs2872507 was most likely associated with RA in European, Japanese and Korean populations. In our study, a chromosomal region including IKZF3 gene and 4,000 bp upstream and 1,500 bp downstream was searched using HapMap and Haploview, and all SNPs identified in Chinese Han population with MAF of 10% or greater were included in the algorithm. Among these tagSNPs, we selected rs12150079, rs9909593 and rs907091 as candidate SNPs, which have not been analyzed in SLE before. We also selected rs2872507 as a candidate SNP as its MAF in Chinese Han population is over 10% and it had been demonstrated earlier to be associated with RA in European, Japanese and Korean populations (the other three, rs9913957, rs8076347 and rs8079075, were with low MAF = 0% in Chinese Han population).

Three newly identified variants in the 11q23.3 region were shown independent association with SLE by performing GWAS on Chinese Han populations from Hong Kong and Anhui. [20] It was shown that they had little LD with variants found in previous studies, suggesting complex biological mechanisms for the involvement of this region in SLE pathogenesis. So it would be important that focusing on the variants in our study with proven association with SLE from the previous work via meta-analysis and further replication in independent cohorts. Additionally, several SNPs (such as rs9913957, rs8076347 and rs8079075) in IKZF3 were previously reported as risk loci for SLE in other ancestries, but these SNPs are all with low MAF (0%) in Chinese Han population. More work will be required to extensively evaluate the role of the rare variants. Although we identified a strong association between rs907091 in the IKZF3 gene and SLE, we still could not determine the potential causal variants in LD with it. Better understanding whether the SNP is functionally relevant or is merely correlated with other unexamined causal polymorphisms will require mechanistic and fine-mapping experiments. Fine mapping and resequencing of this region in Chinese Han population are needed if researchers are to more precisely refine this association and determine the loci associated with risk.

We regret to analyze the few tagSNPs because of the limited working condition and analysis capacity and show the unsatisfactory results and conclusions, which may be caused by the insufficient samples. Expanding the sample size of SLE and analyzing more SNPs of IKZF3 will be an emphasis in our future work. Another limitation is that we only analyzed the SNP with MAF>10% in Chinese Han population. It is possible that some variants with minor allele frequencies <10% also play a role in SLE risk but were not detected.

In conclusion, a strong association between rs907091 in IKZF3 gene and SLE was identified in our study. The CC genotype and C allele of rs907091 were confirmed as protective factors to SLE. Moreover, we also believe that further studies are required to determine whether rs12150079 and haplotype GGCG could influence SLE. These data should be useful to the improvements of the diagnosis, prognosis and therapy for SLE.
